# Antimicrobial Activity Evaluation on Silver Doped Hydroxyapatite/Polydimethylsiloxane Composite Layer

**DOI:** 10.1155/2015/926513

**Published:** 2015-10-04

**Authors:** C. S. Ciobanu, A. Groza, S. L. Iconaru, C. L. Popa, P. Chapon, M. C. Chifiriuc, R. Hristu, G. A. Stanciu, C. C. Negrila, R. V. Ghita, M. Ganciu, D. Predoi

**Affiliations:** ^1^National Institute for Materials Physics, P.O. Box MG 07, 077125 Magurele, Romania; ^2^National Institute for Laser, Plasma and Radiation Physics, 409 Atomistilor Street, P.O. Box MG 36, 077125 Magurele, Romania; ^3^Faculty of Physics, University of Bucharest, 405 Atomistilor Street, P.O. Box MG1, 077125 Magurele, Romania; ^4^Horiba Jobin Yvon SAS, 16-18 Rue du Canal, 91165 Longjumeau Cedex, France; ^5^Microbiology Department, Faculty of Biology, University of Bucharest, 1–3 Portocalelor Lane, Sector 5, 77206 Bucharest, Romania; ^6^Earth, Environmental and Life Sciences Section, Research Institute of the University of Bucharest, 1–3 Portocalelor Lane, Sector 5, 77206 Bucharest, Romania; ^7^Center for Microscopy-Microanalysis and Information Processing, University Politehnica of Bucharest, 313 Splaiul Independentei, 060042 Bucharest, Romania

## Abstract

The goal of this study was the preparation, physicochemical characterization, and microbiological evaluation of novel hydroxyapatite doped with silver/polydimethylsiloxane (Ag:HAp-PDMS) composite layers. In the first stage, the deposition of polydimethylsiloxane (PDMS) polymer layer on commercially pure Si disks has been produced in atmospheric pressure corona discharges. Finally, the new silver doped hydroxyapatite/polydimethylsiloxane composite layer has been obtained by the thermal evaporation technique. The Ag:HAp-PDMS composite layers were characterized by various techniques, such as Scanning Electron Microscopy (SEM), Glow Discharge Optical Emission Spectroscopy (GDOES), and X-ray photoelectron spectroscopy (XPS). The antimicrobial activity of the Ag:HAp-PDMS composite layer was assessed against *Candida albicans* ATCC 10231 (ATCC—American Type Culture Collection) by culture based and confirmed by SEM and Confocal Laser Scanning Microscopy (CLSM) methods. This is the first study reporting the antimicrobial effect of the Ag:HAp-PDMS composite layer, which proved to be active against *Candida albicans* biofilm embedded cells.

## 1. Introduction

One of the major problems encountered in modern medicine is to find new materials that can be integrated in the human body without being rejected by the host tissue. For this purpose, in the recent years, researchers worldwide have focused on the development of novel hybrid materials, by combining the polymer science and nanotechnology, for different biomedical applications, such as drug delivery [1–4] or biomimetic implants [1, 5–9]. In order to be successfully integrated in the human body, the nanoengineered materials must have specific properties, like a good biocompatibility and biodegradability.

Hydroxyapatite (HAp), with the chemical formula Ca_10_(PO_4_)_6_(OH)_2_, belonging to the family of calcium phosphate ceramics is a good candidate for various biomedical applications. Due to its similarity to the mineral phase of human bone tissue and its outstanding properties, such as biocompatibility, bioactivity, osteoconductivity, porosity, and long degradation times [10–13], it has been widely used in orthopedic surgeries as coating material for hip and knee prosthesis or for bone reconstructions [13, 14]. It has been also used in dentistry, as filler in dental prosthesis [13, 14]. However, previous studies have shown that patients with implants coated with pure hydroxyapatite are more likely to develop microbial biofilm associated infections [10, 15]. The biofilm associated infections are implicated in the etiology of 80% of human infections [8], being characterized by slow onset, middle intensity symptoms, chronic evolution, and resistance to antibiotic treatment [16], requiring complex multidrug treatment strategies [17]. One of the proposed solutions for this problem was to embed silver (Ag) nanoparticles in the structure of HAp.

It is well known that silver exhibits high antimicrobial activity against a large number of bacterial and fungal strains [18, 19]. The silver nanoparticles attach to the bacterial cellular membrane causing modification of its permeability, thus disturbing the respiratory function [18, 20]. Furthermore, in order to better improve the biological properties of hydroxyapatite, different polymers can be incorporated in its matrix. This way, both the properties of the host material and of the polymer can be preserved [21, 22]. One of the polymers used for this purpose is polydimethylsiloxane (PDMS), member of the group of polymeric organosilicon compounds. PDMS exhibits a series of very appealing properties, such as high thermal, ultraviolet (UV) and oxidative stability, very low glass transition temperature (−123°C), low surface energy, hydrophobicity, high gas permeability, low permeability to water, low electrical conductivity, and physiological inertness [21, 23], thus making it a good candidate for improving the HAp mechanical properties. According to Mark [24], PDMS is part of a group of promising polymers with low surface energy for biological applications that have the capacity to effectively liberate the absorption of biofoulants. Moreover, recent studies conducted by Brady and Singer [25] have shown that the great freedom of free rotation silicon-oxygen back to bone obstructs the possibility of forming dipolar or hydrogen bonds with complementary functional groups of biofoulants. Lin et al. [26] in recent studies on synthesis and antimicrobial activities of polysiloxane-containing quaternary ammonium salts on bacteria and phytopathogenic fungi have shown that polysiloxanes are especially attractive as they proved particularly high static and dynamic flexibility in many solvents, high permeability, and special surface properties.

In particular, PDMS layers are extensively used in medical implants and biomedical devices becoming a preferred soft substrate for culturing different types of cells [27] due to their biocompatibility [28], nontoxicity toward many species of organisms, and biodegradability [29].

Therefore, it is expected that a hybrid material constituted of silver doped hydroxyapatite and PDMS will exhibit improved mechanical, biological, and antimicrobial properties, thus making it an excellent candidate for numerous biomedical applications.

In this study we reported for the first time the evaluation of antifungal biofilm activity on silver doped hydroxyapatite/polydimethylsiloxane (Ag:HAp-PDMS) composite layer. For these biological studies the silver doped hydroxyapatite/polydimethylsiloxane (Ag:HAp-PDMS) composite layer was formed after the deposition by thermal evaporation technique of three layers of Ag:HAp on a silicon substrate previously coated with a PDMS layer.

The Ag:HAp-PDMS composite layers were investigated by various techniques such as Scanning Electron Microscopy (SEM), Glow Discharge Optical Emission Spectroscopy (GDOES), and X-ray photoelectron spectroscopy (XPS). The antimicrobial and antibiofilm activity of the Ag:HAp-PDMS composite layer were assessed against* Candida albicans* ATCC 10231 strains by culture based and confirmed by Confocal Laser Scanning Microscopy (CLSM) methods.

## 2. Experimental Section

### 2.1. Sample Preparation

#### 2.1.1. Silver Doped Hydroxyapatite (Ag:HAp) Nanoparticles

In order to synthesize the silver doped hydroxyapatite (Ag:HAp) precursors of calcium nitrate [Ca(NO_3_)_2_·4H_2_O, Aldrich, USA], ammonium hydrogen phosphate ((NH_4_)_2_HPO_4_, Wako Pure Chemical Industries Ltd.) and silver nitrate (AgNO_3_, Alpha Aesare, Germany, 99.99% purity) were used. Controlled amounts of ammonium hydrogen phosphate and silver nitrate were dissolved in ethanol. After adding distilled water, the solution was stirred vigorously for 24 h at 40°C. In a separate container, a stoichiometric amount of calcium nitrate was dissolved in ethanol with vigorous stirring for 24 h at 40°C. The Ca-containing solution was added slowly to the P-containing solution and then aged at room temperature for 72 h and further at 40°C for 24 h. The composition ratios in the Ag:HAp (*x*_Ag_ = 0.5) sol were adjusted to have [Ca + Ag]/P as 1.67 [30, 31]. The obtained Ag:HAp nanopowders were treated at 800°C for 6 hours.

#### 2.1.2. Deposition of PDMS Polymer Layer on Commercially Pure Silicium (Si) Disks

The PDMS layers have been produced in atmospheric pressure corona discharge starting from liquid precursors of vinyl terminated polydimethylsiloxane. The details regarding the experimental set-up and the procedure of PDMS layer generation on metallic substrates was presented in [32, 33].

#### 2.1.3. Deposition of Ag:HAp Nanoparticles on a Silicon Substrate Previously Coated with a PDMS Layer

The Ag:HAp (*x*_Ag_ = 0.5) powder treated at a temperature of 800°C for 6 hours has been deposited by thermal evaporation technique as solid layer on a silicon substrate previously coated with a PDMS layer [34]. By this technique the Ag:HAp nanoparticles (source material) are evaporated in vacuum. The vapour particles travel directly to the substrate where they condense to a solid state. A HOCH VACUUM Dresden installation was used under environment conditions. The pressure in the deposition chamber was in the range of 8 · 10^−5^ torr. The time range for a deposition cycle was around 120 min. The substrate was maintained at room temperature and at ground electrical potential. The Ag:HAp powder evaporation temperature was 1100°C. The tungsten boat temperature during the Ag:HAp powder evaporation is in the range of 1178–1205°C. The distance between substrate and boat is 5 cm.

The evaporation time measured during deposition is situated in the range 20 sec for a maximum current intensity of *I* = 75 A and in the range of 15 sec for a maximum current *I* = 80 A. Taking into account the deposition characteristics such as the total amount of HAp totally deposited (in mg) and the substrate-boat distance, the calculated evaporation velocities were *v*_1_ = 0.167 mg/s or *v*_1_ ~ 8.3 nm/s. The calculated thickness of the deposited Ag:HAp: 50% layer, in the experimental conditions presented above, on a silicon substrate is about 480 nm [34]. In the presence of a PDMS layer on the substrate, the Ag:HAp evaporated particles diffuse into the PDMS layer during their travel to the substrate. When the Ag:HAp particles stop in the polymer layer they transfer their energy to the polymer. Thus, the local temperature increases and as the polymer is heated, the thermal condition of a new compound generation is assured. In the following sections, the obtained composite layer is investigated by different methods.

### 2.2. Characterization Methods

The morphology of the material was studied using a Quanta Inspect F Scanning Electron Microscope (SEM). The elemental local analysis of the coatings was performed using an energy dispersive spectroscope (EDS) detector from X-EDS (Energy Dispersive X-Ray Spectroscopy). Operating conditions were an accelerating voltage between 2 and 25 keV (depending on the ratio signal/noise) with samples tilted at 25°C to get the optimal take off angle (30°) allowing a dead time around 20–30% and a collecting time of 90–120 s. The top surface analysis of the samples was studied by Glow Discharge Optical Emission Spectroscopy (GDOES) [35] using a GD Profiler 2 from Horiba/Jobin-Yvon. Glow Discharge Optical Emission Spectroscopy (GDOES) is an essential technique for direct analysis of bulk solids and for elemental surface analysis and depth profiling of thin films and industrial coatings [36]. The X-ray photoelectron spectroscopy (XPS) measurements were performed using a VG ESCA 3 MK II XPS installation (*Ekα* = 1486.7 eV). The vacuum analysis chamber pressure was *P* ~ 3 × 10^−8^ torr. The XPS recorded spectrum involved an energy window *w* = 20 eV with the resolution *R* = 50 eV with 256 recording channels. The XPS spectra were processed using Spectral Data Processor v 2.3 (SDP) software. After Shirley background subtraction, the deconvolution of the XPS curves was conducted by using a fitting procedure based on the summation of Gaussian functions. When irradiated with X-rays, electrons from the inner shells cause the apparition of several peaks with different shapes in the analyzed spectra. Due to the characteristics of the measurement device that can lead to distortions and the ionization process, the peaks can be considered a convolution of Lorentz and Gaussian functions. The Lorentz function is associated with the lifetime broadening described by the uncertainty principle which depicts the relation between the lifetime and the energy of the ejected electrons, whereas the Gaussian function is associated with the measurement process. Theoretically, in some cases, the complexity of the obtained spectra comprises asymmetries caused by photoelectric peaks. Moreover, when selecting a line shape for fitting experimental data, both the background shape and theoretical considerations must be taken into account. Since the energy of the radiation is not monochromatic and the electron analyzer resolution (pass energy) was *R* = 50 eV, the obtained spectra were processed using a Gauss function. Furthermore, it is well known that the Lorentz component increases for spectral lines with high binding energy. As a result, in this case, the Lorentz component is negligible. The hardness (*H*) and Young's modulus (*Y*) of the films were determined by nanoindentation experiments with a Berkovich indenter. The hardness and Young's modulus were extracted from nanoindentation measurements using approach similar to the Oliver and Pharr analysis method [37–39]. In order to avoid substrate interaction effects, the indentation depth is about 10% roughly of film thickness.

### 2.3. Antibiofilm Activity

The antibiofilm activity of the obtained composites carried out against* Candida albicans* (*C. albicans*) biofilms was quantified at 24 hours and 48 hours. In this purpose,* C. albicans* ATCC 10231 (500 *μ*L of 0.5 McFarland microbial suspension in sterile saline obtained from 24 h microbial cultures) was grown on the thin films immersed in 4 mL culture medium (liquid yeast peptone glucose, YPG). At intervals of 24 hours and 48 hours the thin films were removed from the culture medium, washed in sterile saline solution in order to remove the nonadherent yeast cells, introduced into sterile saline (1 mL), and then vortexed for suspending the microbial cells embedded in the biofilm formed on the thin films specimens. This whole procedure was repeated for thin films colonized with fungal biofilms for 48 hours. The density of the microbial suspension obtained by removing the formed biofilms at 24 h and 48 h was measured spectrophotometrically at 620 nm. Duplicate thin films specimens were washed in saline solution, fixed in cold methanol, and prepared for CLSM examination [40]. In this purpose, the specimens were stained for 2 minutes with ethidium bromide and visualized in reflection and fluorescence modes by using a TCS SP confocal microscope, equipped with a 10X HCX PL FLUORITE objective, with a numerical aperture NA of 0.3. An Ar ion laser (488 nm) used to simultaneously acquire both reflection and fluorescence. A lateral resolution of about 600 nm was achieved [41].

## 3. Results and Discussions

**Figure 1:**
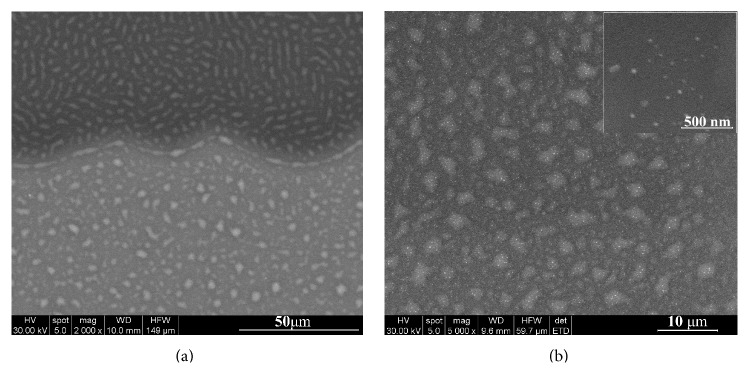
SEM image of (a) interface zone between the Ag:HAp-PDMS composite layer and the Ag:HAp layer; (b) Ag:HAp-PDMS composite layer with two different magnifications.

The morphology and the features of the composite layers formed after the thermal evaporation of the Ag:HAp nanoparticles and their consecutive deposition on a PDMS layer/Si substrate were studied by Scanning Electron Microscopy (SEM). The Ag:HAp based coating covers entirely the Si substrate surface (with a diameter of 20 mm) while the PDMS layer was deposited only on a circular area with a diameter of 10 mm. An image of the interface zone between the Ag:HAp-PDMS composite layer and the Ag:HAp layer is presented in Figure 1(a). It can be observed that the polymer acts as a matrix for the Ag:HAp coating. Both the Ag:HAp-PDMS composite layer and the Ag:HAp layer are compact, homogeneous, and with no cracks. Two high resolution SEM images of the Ag:HAp-PDMS composite layer are presented in Figure 1(b).

**Figure 2:**
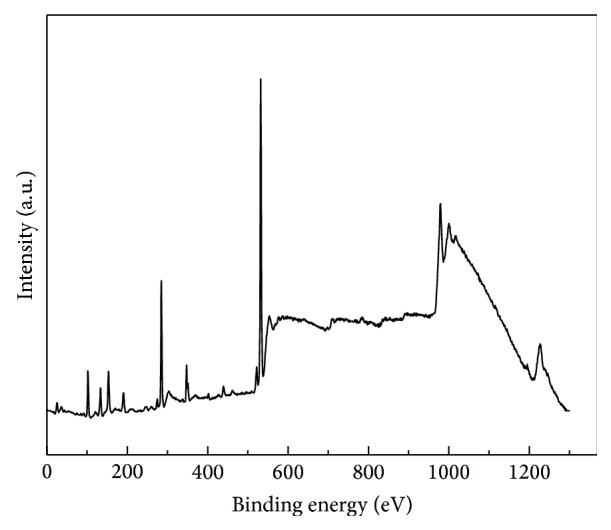
Survey spectra of Ag:HAp-PDMS composite layer.

**Figure 3:**
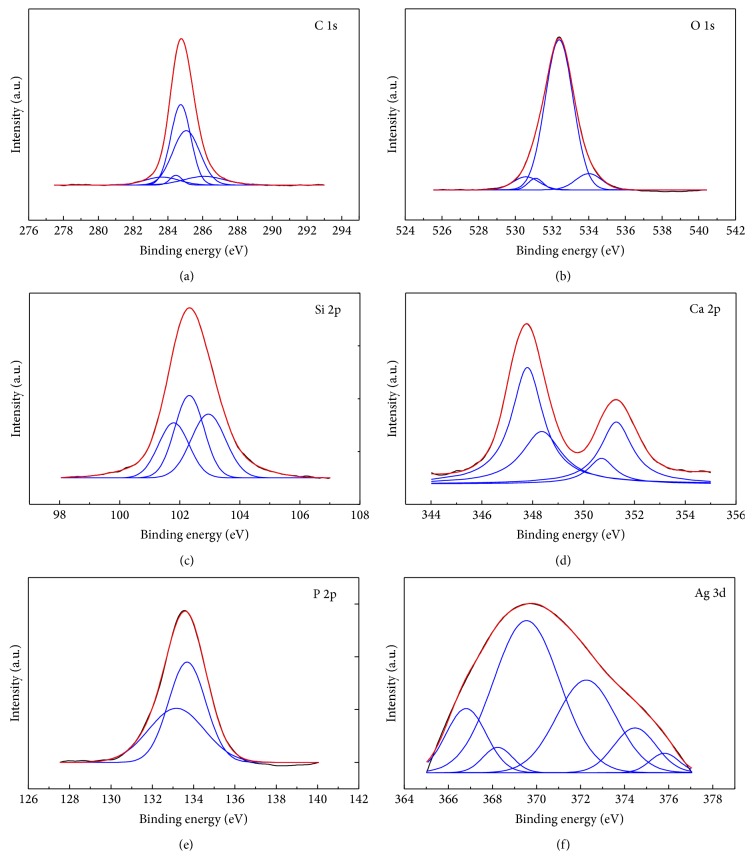
High resolution XPS spectra of C (1s) XPS peaks (a), O (1s) XPS peaks (b), Si (2p) XPS peaks (c), Ca (2p) XPS peaks (d), P (2p) XPS peaks (e), and Ag (3d) XPS peaks (f) of the Ag:HAp-PDMS composite layer.

In order to investigate the elements components and the chemical modification generated on PDMS and its effect on silver doped hydroxyapatite film formation on the surface of PDMS, X-ray photoelectron spectroscopy (XPS) measurements were performed. XPS high resolution spectra of the C 1s, Si 2p, O 1s, Ca 2p, P 2p, and Ag 3d regions were obtained.  Figure 2 displays the survey spectra of Ag:HAp-PDMS composite layer. The measured binding energy (*E*_*B*_) scale was referenced to a C 1s at the *E*_*B*_ value of 284.8 eV [42]. The accuracy for *E*_*B*_'s assignments is ±0.2 eV. XPS spectral peaks for C 1s (Figure 3(a)) were deconvoluted into five Gaussian components attributed to C 1s binding energies of the C–C, C–H, and C=O. In the opinion of Serra et al. [43] the peak positioned at 284.4 eV, corresponds to C–C and C–H bonds. The peak at 284.7 eV can be designated to C–C group which is the primary chemical band of pure PDMS [44, 45]. On the other hand, the peak at 285 eV related to C–C bond represents a part of adsorbed carbon on sample surface together with the C–C bond in PDMS [46]. By Prieto et al. [47] the peak at 285.0 eV is assigned to C–C and C–H hydrocarbon bonds. The peak at 286.1 eV can be assigned to either (O–C) or carbonyl (O–C=O) groups, respectively [48, 49]. According to Prieto et al. [47] the peak at 286.1 eV is due to C–OH and C–O bonds. The peak positioned at 283.4 eV could correspond to C-metal bonds, showing a chemical interaction between Ag and contaminants from surface layer.

Figure 3(b) shows the high resolution XPS spectra of oxygen O 1s for Ag:HAp-PDMS composite layer. The O 1s photoelectron peak was deconvoluted into four Gaussian components. According to previous studies presented by Zemlyanov et al. [50], the first component placed at 530.5 eV can be assigned to the lattice oxygen (Ag–O–Si) of the Ag:HAp-PDMS thin film. According to the literature data on the oxygen and hydroxyl adsorption on Ag [51], the desorption temperature of the observed O 1s peaks allows attributing the peak at 531.1 eV to surface OH (O_ads_^*γ*−^) groups. This result is also in good agreement with previous studies of Kawabe et al. [52] believing that two oxygen species, that is, O^−^ and OH^−^, may be included in the resolved peak at 531.1 eV. The peak at 532.3 eV may be attributed to the oxygen linked to a phosphorous atom as in PO_4_^3−^ ions [53]. In conformity with previous XPS results reported by Jeon and Kang [54], Carroll et al. [55], and Xu and Khor [53], the small component at 534.0 eV can be assigned to absorbed oxygen (Si–O) and O–C–O bonds.  Figure 3(c) shows the high resolution XPS peak of Si 2p core level. Si 2p spectra can be separated into three Gaussian components.

The peak at 101.8 eV can be attributed to Ag–O–Si linkages [56]. According to [53] and Mekki et al. [57], the binding energy of SiO_4_^4−^ was centred at 102.3 eV. The peak at 103.1 eV was allocated to Si–O–Si linkages [58]. The Ca 2p spectrum of the Ag:HAp-PDMS composite layer exhibits a well-resolved doublet with a Ca 2p_3/2_ component and a Ca 2p_1/2_ component (Figure 3(d)).

The XPS spectral peaks for Ca 2p_1/3_ were decomposed into a two Gaussian components at around 350.6 and 351.2 eV [53]. The XPS spectral peaks for Ca 2p_3/2_ were decomposed into two Gaussian components. The first peak located at 347.8 eV and the second peak located at about 348.3 eV show that the calcium atoms are bonded with a phosphate group (PO_4_^3−^) [53]. After the deconvolution data processing, the P 2p photoelectron line consists of two Gaussian components that were established at 133.1 eV and 133.7 eV. These values are in the range of binding energies determined for hydroxyapatite [59, 60]. The high resolution XPS spectra and curve-fitting results of phosphorous P 2p for Ag:HAp-PDMS composite layer are exhibited in Figure 3(e). Stoica et al. showed [61] that the P 2p photoelectron line consists of one single peak at *E*_*B*_ position of 133.4 eV. The high resolution XPS spectra of the Ag 3d region obtained from Ag:HAp-PDMS composite layer is presented in Figure 3(f). The Ag 3d spectrum exhibits a broad doublet with an Ag 3d_5/2_ and Ag 3d_3/2_ component. The XPS spectral peaks for Ag 3d were decomposed into six Gaussian components located at 366.7 eV, 368.2 eV, 369.5 eV, 374.2 eV, 374.4 eV, and 375.8 assigned to silver ions in Ag^+^ and Ag^2+^, Ag_2_O (Ag^+^), and Ag–O and Ag–Ag, in good agreement with literature values [62–65].

XPS results revealed that the silver from Ag:HAp-PDMS composite layer was assigned to Ag^+^, Ag^2+^, Ag_2_O (Ag^+^), Ag–O, and Ag–Ag silver ions. These results showed that there was some kind of interaction between Ag from Ag:HAp and polydimethylsiloxane. On the other hand, the GDOES analysis has shown that during the deposition process there are some interactions of Ag:HAp particles with the polymer and thus the formation of a new composite material. Moreover, this study develops a novel and facile method to produce a new composite based on Ag (Ag:HAp-PDMS composite layer) which can be used for large-scale applications. The reproducibility, applications, and the price of the new products are not limited due to use of expensive precursors, such as tetraethoxysilane or due to the complicated synthesis route.

**Figure 4:**
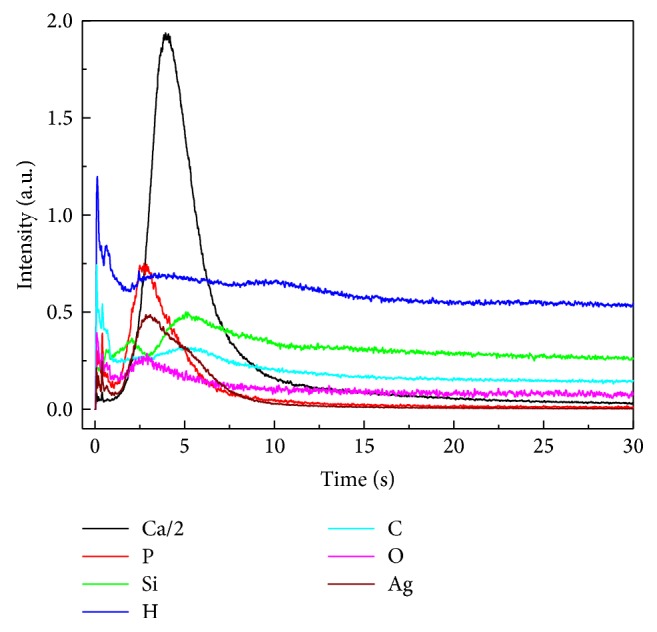
GDOES spectra of the Ag:HAp-PDMS composite layer formed on the Si substrate.

The GDOES spectra of the Ag:HAp-PDMS composite layer, presented in Figure 4, indicate that the Si, O, C, H (atoms specific to a PDMS layer) [66], P, Ca, Ag, and O (atoms specific to a Ag:HAp layer) [67] depth profile curves have similar temporal behavior [34]. There is not any sharp increasing or decreasing in their depth profile curves as in the case of multilayer analysis [68]. Therefore, in correlation with the SEM and XPS analysis, the GD results indicate that during the thermal deposition process the Ag:HAp particles interact with the PDMS layer previously deposited on the silicon substrate determining the formation of a composite material.

The hypothesis of Si and O atoms redistribution in the bulk of the Ag:HAp-PDMS composite and their possible involvement in the Si–O, Si–O–Ag, and Si–O–P bonds formation is also sustained by their depth profile curves in comparison with the Si and O depth profile curves observed in a PDMS layer [66] presented in Figure 4. The Si depth profile curve decreasing only after the Ca depth profile curve (and all the other elements) drop down can indicate not only the silicon involvement in the silicon oxides structures, Si–O–Ag, or Si–O–P bonds formation but also the incorporation of SiO_4_^4−^ ions in the hydroxyapatite doped with silver structure by the mechanism of SiO_4_^4−^/PO_4_^3−^ ions substitution as was suggested by the XPS studies presented above.

The hardness (*H*) and Young's modulus (*Y*) of our Ag:HAp layer on a silicon substrate are 2.7 GPa and 98 GPa and 2.6 GPa. For Ag:HAp-PDMS composite layer on a silicon substrate the *H* and *Y* values are 3.28 GPA and 85 GPa, respectively. For Ag:HAp nanoparticles deposited on a silicon substrate previously coated with a PDMS layer (Ag:HAp-PDMS) the *Y* modulus is lower than that for Ag:HAp layer while the *H* modulus is greater than that for Ag:HAp layer. The values of *H* and *Y* moduli are influenced by the presence of PDMS. The values obtained for *H* and *Y* moduli are in agreement with existing literature for crystalline samples [69].

The fungal infections represent an emerging medical problem, particularly in immunocompromised patients [70]. Fungal pathogens can exhibit various mechanisms of resistance to common antifungals [71]. An important role in the evolution and treatment of fungal infections is attributed to microbial biofilms developed on natural tissues or artificial devices [72], according to NIH (National Institute of Health) [73].

**Figure 5:**
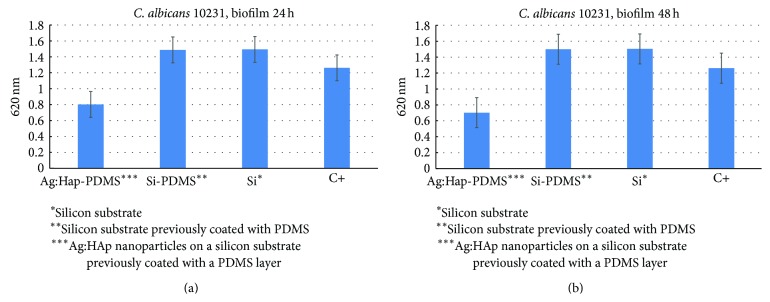
The graphic representation of the fungal biofilm development on different substrates, as revealed by the density of the microbial suspension recovered from the biofilms adhered on the tested specimens.

The biofilm resistance is phenotypic and was also called tolerance [74]. One of the strategies employed to overcome this challenging problem is the design of novel biomaterials with improved resistance to microbial colonization.* C. albicans* represents the most prevalent fungal species involved in biofilm associated infections, either superficial or systemic ones [75–80]. Therefore, we have chosen to assess the antibiofilm activity of the novel composite layers against this fungal pathogen. The obtained composite layers have been proved to exhibit superior resistance to fungal colonization as compared with single materials used to obtain the composite (Figure 5). Concerning the dynamics of the fungal biofilm, it can be observed that the inhibition of the fungal biofilm development is gradually increasing from 24 h to 48 h, as demonstrated by the lower absorbance values obtained for 48 h biofilms harvested from the Ag:HAp-PDMS composite layers (Figure 5(b)), as compared to those obtained for 24 h biofilms recovered from the same substratum (Figure 5(a)). Furthermore, on the rest of the tested materials, that is, Si-PDMS layer and Si substrate, no significant differences have been noticed for the biofilms developed on these materials after 24 h and 48 h.

These results could be explained by the fact that the silver incorporated in the HAp-PDMS matrix is gradually released and exhibits its antimicrobial effect on a prolonged period of time, preventing both the early phase of the fungal biofilm development in which yeast cells adhere to the substrate surface and undergo hyphal growth and the development of the mature biofilm, consisting of a mixture of yeast and hyphal elements forming a complex network encased in a self-produced extracellular material [81, 82].

**Figure 6:**
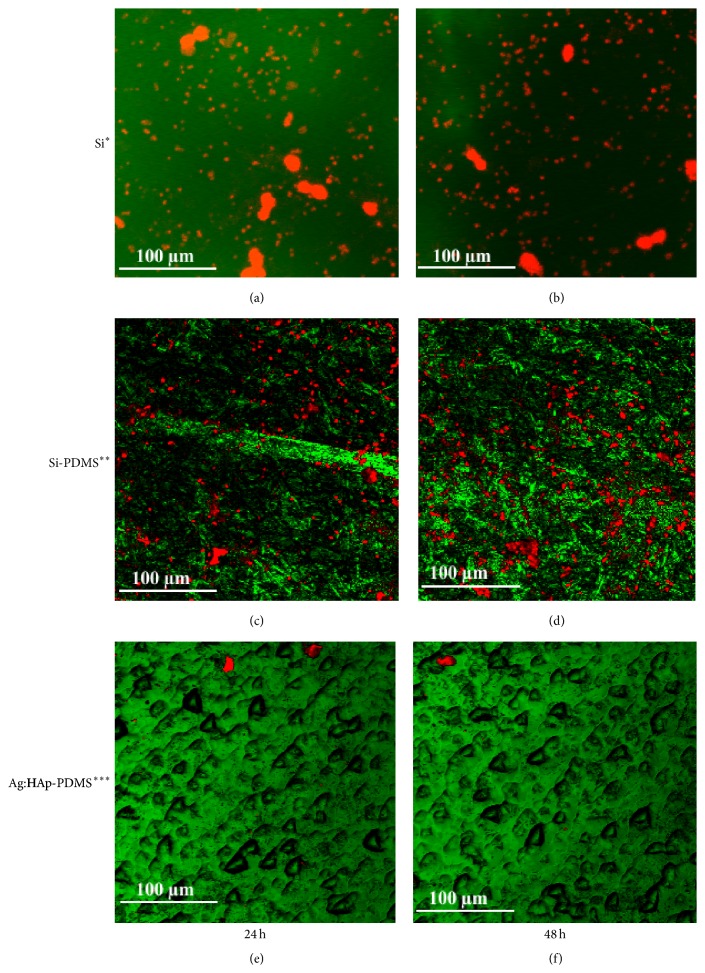
CLSM images of* C. albicans* biofilm stained with ethidium bromide developed on different substrata at 24 h and 48 h (^*∗*^silicon substrate, ^*∗∗*^silicon substrate previously coated with PDMS, and ^*∗∗∗*^Ag:HAp nanoparticles on a silicon substrate previously coated with a PDMS layer).

The culture based results were confirmed by CLSM images revealing the fungal biofilms developed on different surfaces. Visualization of control biofilms revealed that the fungal biofilm stained in red by the ethidium bromide better developed on Si substrate and Si-PDMS layer, both at 24 h (Figures 6(a) and 6(c)) and 48 h (Figures 6(b) and 6(d)). In exchange, visualization of the biofilm structure developed on the Ag:HAp-PDMS composite layer revealed an important decrease in biofilm development (Figures 6(e) and 6(f)). This suggests the diffusion of Ag ions from the composite, exhibiting a prolonged antimicrobial and antibiofilm activity.

**Figure 7:**
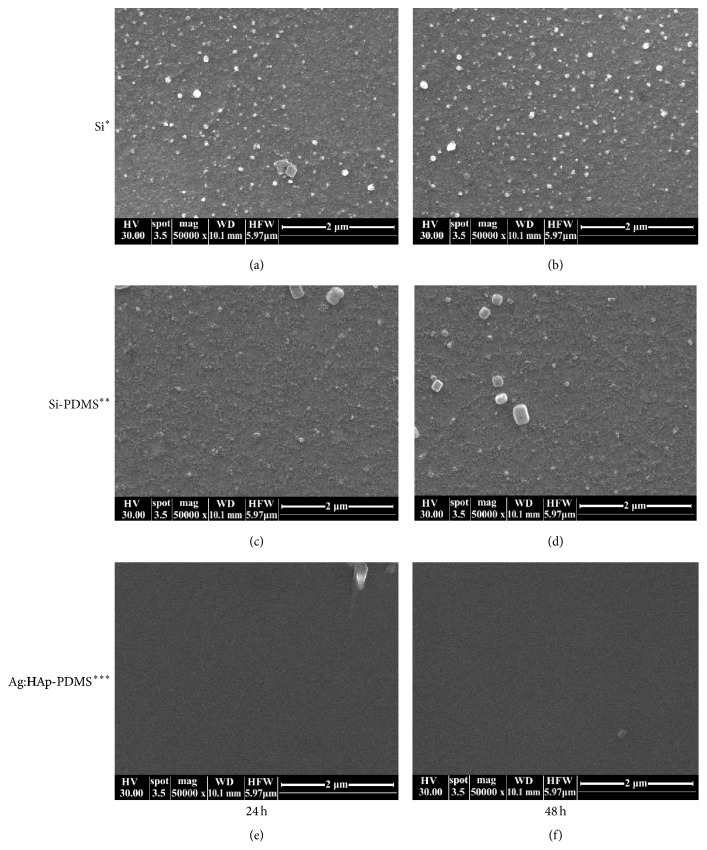
SEM photographs of* C. albicans* biofilm stained with ethidium bromide developed on different substrata at 24 h and 48 h (^*∗*^silicon substrate, ^*∗∗*^silicon substrate previously coated with PDMS, and ^*∗∗∗*^Ag:HAp nanoparticles on a silicon substrate previously coated with a PDMS layer).

The growth of* C. albicans* biofilm on various substrates was also observed by Scanning Electron Microscope (SEM).  Figure 7 presents photographs after 24 h and 48 h from microbial contamination with* C. albicans*. SEM observations have revealed different growth rates of the* C. albicans* biofilm on the silicon substrate, on the silicon substrate which was previously coated with PDMS and on the layer formed by deposition of Ag:HAp nanoparticles on a silicon substrate previously coated with a PDMS layer.

Also, it can be seen that the incubation time influenced the growth of* C. albicans* biofilm on different substrates. In the SEM images it is observed that the fungal biofilms have developed significantly on Si substrate (Figures 7(a) and 7(b)) and Si-PDMS layer (Figures 7(c) and 7(d)) both after 24 h and after 48 hours, matching the results obtained from CLMS images (Figure 6). A significant decrease of biofilm development was observed on the Ag:HAp-PDMS composite layer (Figures 7(e) and 7(f)).

*C. albicans* is an important fungus responsible for systemic infections in humans. The increases of morbidity and mortality caused by* C. albicans* are very high and researchers worldwide are searching new alternative therapies and new drugs to reduce these infections. In conformity to Luo et al. [83] new prophylactic and therapeutic strategies are urgently needed to prevent fungal infection. According to recent studies conducted by Taglietti et al. [84] many* Candida* infections involve the formation of biofilms on implanted devices such as indwelling catheters or prosthetic heart valves. On the other hand, infections by* C. albicans* hospital acquired have become a cause of major health concerns. Silver nanoparticles represent a new generation of antimicrobial materials used as coatings. The silver nanoparticles have the ability to act locally, having a bactericidal effect. Nevertheless, the use of silver nanoparticles may pose a potential risk of cytotoxic effects on eukaryotic cells. To decrease a potential risk of cytotoxic effects of silver nanoparticles on eukaryotic cells, the composite materials endowed with bactericidal abilities have been achieved by adding the silver nanoparticles into various types of biomaterials.

Recent studies conducted by Ciobanu et al. [85, 86] on silver doped hydroxyapatite nanoparticles have demonstrated a good antimicrobial activity for all the studied concentration of the silver in the samples. Moreover, recent studies on thin solid films of silver doped hydroxyapatite prepared by sol-gel method [67] have also demonstrated a good antimicrobial activity against* Escherichia coli* and* Staphylococcus aureus* bacteria. It has thus been shown that the Ag:HAp thin films could be used to cover the surface of implantable medical devices. The results obtained in the studies presented in this paper demonstrate that, on the Si-PDMS layer and Si substrate, the microbial activity increases after 24 h and 48 h for biofilms developed on these materials. On the biofilm structure developed on the Ag:HAp-PDMS composite layer, the microbial activity decreases significantly for the surveyed time intervals. On the other hand, the CLMS technique used for the characterization of biofilm structure allowed us to obtain important information regarding the role of Ag ions in prolonged antimicrobial and antibiofilm activity.

In this context, novel hydroxyapatite doped with silver/polydimethylsiloxane (Ag:HAp-PDMS) composite layers is a primary target for new anti-infective strategy that could lead to a decrease in contamination with fungi and bacteria of medical devices by coating their surfaces. This study is part of the global research on the development of innovative biomaterials that possess anti-infective properties and technologies that allow the creation of bactericidal biomaterial surfaces [87, 88].

Fungal infections caused by* C. albicans* are determining factors for morbidity and mortality in both the immunocompetent and the immunocompromised critically ill patients (e.g., Human immunodeficiency virus infection/acquired immunodeficiency syndrome (HIV/AIDS), cancer chemotherapy, and organ or bone marrow transplanted [89, 90]). According to [91]* C. albicans* efficiently adheres to polystyrene, polyvinylchloride, silicon, and polycarbonate, colonizing on their surfaces. Moreover, Shinde et al. [91] showed that* Candida* biofilms are highly resistant to drugs such as fluconazole. Furthermore, it was observed that* C. albicans* biofilm formation was dependent both on the microbial strain and on the substrate. Recent studies [91] on* C. albicans SRTCC-06* isolated from the feces of a patient admitted to the tertiary care hospital for treatment of diarrhea and* C. albicans SRTCC-11* isolated from a patient diagnosed with vaginitis have shown that the strain variation has a more pronounced effect on adhesion than the involved substrates. On the other hand, Shinde et al. [91] showed that the ability to adhere to prosthetic devices also depends on the properties of the cells and the substrate plays an important role in the biofilm formation of* C. albicans*. El-Azizi and Khardori [92], in their studies on “Factors influencing adherence of* Candida spp*. to host tissues and plastic surfaces,” have shown that various properties including surface-free energy and roughness of the substrates may influence adhesion and biofilm development. Their results were confirmed by Raut et al. [93] in her studies on “Cell surface hydrophobicity and adhesion: a study on fifty clinical isolates of* Candida albicans.*”

Considering that the fourth most common cause of nosocomial BSIs is the* Candida* strains [94], our data demonstrate that the substrates based on Ag:HAp-PDMS composite give a new perspective on the development of new prosthetic surfaces.

Our study was focused on creating a substrate that prevents the development of* C. albicans* biofilm thus creating an alternative solution to the issue of prosthesis-associated* C. albicans* infections. Results indicated an important decrease in biofilm development of* C. albicans* on Ag:HAp-PDMS composite layer and these composite layer based on Ag:HAp-PDMS may be used as potential prosthetic materials. Therefore we can say that the nature of the substrate can lead to a decrease of the* C. albicans* virulence.

## 4. Conclusions

Using XPS and GDOES spectral technique we have investigated the physicochemical processes that take place by the interaction of Ag:HAp particles with the PDMS layer. The XPS measurements showed that the physical procedure used for the generation of the Ag:HAp-PDMS composite layer allows the formation of SiO_4_^4−^ ions. The SiO_4_^4−^ ions can be incorporated into the Ag:HAp structures by substitution of PO_4_^3−^ ions from the structure of Ag:HAp. We suppose that after the condensation of the Ag:HAp particles on the substrate, due to the heating of the SiO_2_ network present into the polymer bulk, some SiO_4_^4−^ ions are generated. The crystalline structure of the Ag:HAp was also evidenced.

The GDOES depth profiling curves of the Ag:HAp-PDMS composite layer indicate that its composition is homogeneous which can be explained by the formation of the Si–O–Ag, respectively, Si–O–P bonds and Si involvement in Ag:HAp structure.

The development of* C. albicans* biofilm, both the initial adherence phase and the mature biofilm, was strongly inhibited by the Ag:HAp-PDMS composite layer, as shown by viable cell counts, CLSM, and SEM examination. Taking into account the important implications of* C. albicans* in human biofilm associated infection, the new composite materials could be of a great interest in the biomedical field, for the design of coatings that prevent or lag the fungal biofilm development.
